# The effect of credence attributes on willingness to pay a premium for organic food: A moderated mediation model of attitudes and uncertainty

**DOI:** 10.3389/fpsyg.2023.1087324

**Published:** 2023-02-17

**Authors:** Hong Huo, Xinyu Jiang, Chunjia Han, Sheng Wei, Dingyao Yu, Yang Tong

**Affiliations:** ^1^Department of Management, Harbin University of Commerce, Harbin, China; ^2^Department of Management, Birkbeck, University of London, London, United Kingdom; ^3^Department of Mechanical and Power Engineering, Zhengzhou University, Zhengzhou, China

**Keywords:** organic food, credence attribute, food safety, eco-friendliness, willingness to pay a premium, attitude, uncertainty

## Abstract

**Objective:**

With consumers’ concerns about food safety and the environment growing, the interest in organic food has increased. However, due to the late start of the organic food market in China, the market size of the Chinese organic food industry is still relatively small. This study aims to examine whether organic food credence attributes have an impact on consumers’ attitudes and willingness to pay a premium (WTPP), in order to provide valuable information to facilitate the development of the organic food market in China.

**Methods:**

A questionnaire survey was conducted with 647 respondents in China. Structural equation modeling (SEM) was utilized to verify the model and test the relationships among the constructs.

**Results:**

SEM analyses showed that credence attributes stimulate consumers’ attitudes and increase consumers’ WTPP. Utilitarian attitudes and hedonistic attitudes play a partially mediating role in the relationship between credence attributes and WTPP. Uncertainty negatively moderates the role between utilitarian attitudes and WTPP, while it positively moderates the role between hedonistic attitudes and WTPP.

**Discussion:**

The findings reveal the motivations and barriers for Chinese consumers to purchase organic food at a premium, providing a theoretical basis for companies to gain a deeper understanding of consumer groups and develop organic food marketing strategies.

## 1. Introduction

With rising consumer preference for food that is perceived as healthy, high quality, safe, and eco-friendly, organic food is being pursued by an increasing number of Chinese consumers. Data show that organic food sales in China reached approximately RMB 951 billion in 2021, an increase of 18.3% compared to 2020 (China, 2022). Organic consumption has become a new trend in China. However, there is a clear gap between consumers’ intentions and behavior in the decision-making process related to purchasing organic food (Sultan et al., 2020; Bernabéu et al., 2022). In other words, although consumers have positive intentions to purchase organic food, their actual purchase frequency and payment amount do not match their intentions, and the key reason for this mismatch is that consumers consider the price of organic food too high. The price premium for organic food has become a major obstacle in the purchasing decision process for consumers (Kim et al., 2021; Liu and Sam, 2022). Therefore, increasing consumers’ willingness to pay a premium (WTPP) is important to promote the development of China’s organic food market.

The motivations for organic food consumption have been extensively explored in previous literature. Studies have found that consumers’ motivations for purchasing organic food include economic reasons (e.g., price), social/cultural reasons (e.g., social status), personal reasons (e.g., egoistic and altruistic values), and product reasons (e.g., attributes such as health and safety; Dorce et al., 2021; Galati et al., 2022; Knaggs et al., 2022; Wei et al., 2022). In particular, concern for health, environmental protection, food safety, and taste have been identified as the main purchase motivations for organic food (Hemmerling et al., 2015). As organic food is a credence product and its product information is harder to obtain, credence attributes play an important role in influencing consumers’ organic food purchasing behavior (Giampietri et al., 2018). The existing studies on the credence attributes of organic food mainly focus on two aspects. One is to investigate the influence of credence attributes on consumers’ perception of quality and value (Lee and Hwang, 2016; Konuk, 2019). Scholars believe that the overall quality and value perception is based on consumers’ evaluation of credence attributes of organic food, and consumers with a positive evaluation of the credence attributes have a higher perception of the quality and value of organic food. The other is based on consumers’ psychological decision-making process. The influence of credence attributes on consumers’ purchase attitudes and intentions was analyzed, and it was found that credence attributes such as health, safety, and environment-related factors of organic food all lead to positive attitudes and purchase intentions (Szolnoki and Hauck, 2020; Galati et al., 2022). These studies on credence attributes have provided insights into consumers’ motivations for purchasing organic food. However, the existing studies have focused more on the relationship between credence attributes and willingness to buy (WTB) than on willingness to pay a premium (WTPP; Lee and Hwang, 2016; Lee et al., 2019). Given that organic food is a credence good and that there is a degree of premium, the relationship between credence attributes and WTPP needs to be explored in more depth.

In terms of barriers to organic food consumption, several studies have attempted to explain the constraints in consumers’ decisions related to organic food purchases through moderating variables, such as scepticism about organic certification (Moruzzo et al., 2020), limited availability (Rabadán et al., 2020), and price (Rödiger and Hamm, 2015). Due to the late start of the organic food market in China, there is still a significant information asymmetry between organic food sellers and consumers (Zhao et al., 2019), which creates uncertainty about the benefits of organic food and to some extent weakens consumers’ intention to buy organic food (Talwar et al., 2020). Therefore, addressing consumers’ uncertainty regarding organic food may be of great significance in promoting organic food consumption. Nonetheless, there have been scarce studies emphasizing the association of uncertainty with organic food consumption.

China is the fourth largest organic food market after the United States, Germany, and France (China, 2022). The Chinese organic food market represents a great opportunity not only for Chinese producers but also for national exporters of organic food (Pedersen et al., 2022). Given the situational background of the organic food market in China, it is necessary to investigate and fully understand the reaction of Chinese consumers related to organic food. Therefore, this study explores the content of food safety and eco-friendliness, two key credence attributes that promote organic food consumption (Hemmerling et al., 2015), based on a survey from China. The theoretical model of the stimulus-organic-response (S-O-R) model is then used to examine the relationship between the credence attributes, attitudes along the dimensions of utilitarianism and hedonism, and WTPP. Uncertainty is added as a moderating variable to delve into the barriers in the formation of consumers’ purchase decisions. This study is of particular importance because it provides both new, practice-relevant empirical evidence in the field of organic food consumption and useful information for organic food producers, retailers, and exporters to gain a deeper understanding of consumer motivations. In addition, this study could greatly assist relevant regulators and policy-makers in their efforts to improve and expand the organic food market.

## 2. Theoretical background and hypotheses

The stimulus-organism-response (S-O-R) model has its origins in environmental psychology, which assumes that external stimuli trigger internal cognitive and psycho-affective changes in individuals, resulting in certain behavioral outcomes (Darby and Karni, 1973). Stimuli are specific cues that affect an individual’s internal state, i.e., various information that is internal or external, controllable or uncontrollable, in response to the quality of the product. The organism is the emotional and cognitive state of an individual when exposed to external stimuli, including attitudes, emotions, perceptions, and beliefs that typically mediate the relationship between stimuli and responses. Reactions are the final behavioral outcomes of individuals, including intentions, preferences, and behaviors, which may be positive or negative (Ahmed et al., 2021).

The S-O-R model has been successfully used in the field of organic food consumption. For example, Wang et al. (2021) adopted SOR theory and found that organic appeal advertising positively influences intrinsic motivation, increasing consumers’ purchase intention toward organic milk. According to the S-O-R model, Sultan et al. (2021) found that marketing communication channels for organic food and perceived organic food value (S) had a positive effect on utilitarian and hedonistic attitudes (O) and further influenced consumers’ behavioral intentions (R). Liu and Zheng (2019) used health concerns, environmental concerns, and food safety as external stimuli, organic cognition as the organism, and purchase intentions as the response, confirming the significant role between consumers’ food safety, health, environmental concerns, and their purchase intentions. Although the S-O-R model has shown to be useful in predicting intentions and behaviors related to organic foods (Hempel and Hamm, 2016; Kim et al., 2021; Liang and Lim, 2021), most studies have included purchase intentions as a response in the S-O-R model, with less attention given to consumers’ WTPP for organic food. This study uses consumers’ WTPP as a response, which helps to complement the theoretical research on S-O-R models in the organic food field. Furthermore, although some past studies have emphasized the importance of credence attributes for predicting consumers’ behavioral intentions toward organic food (Lee and Hwang, 2016; Kushwah et al., 2019), there is a lack of studies that combine credence attributes with S-O-R. Lee and Yun (2015) suggested that future research should extend the S-O-R model and measure how other causal and moderating factors affect behavioral intentions. We further extended the S-O-R model by adding uncertainty as a new moderating variable, according to the study by Lee and Yun (2015).

In conclusion, given the importance of S-O-R in explaining the relationship between external factors and consumer responses in previous studies, this study applies S-O-R theory to explain the effect of credence attributes on willingness to pay a premium for organic food by defining stimuli as food safety and eco-friendliness, organisms as utilitarian and hedonistic attitudes, responses as consumers’ willingness to pay a premium, and introducing uncertainty as a moderating variable. On the one hand, the change in consumers’ attitudes and their willingness to pay a premium in their natural state, stimulated externally by the credence attributes of organic food, fits well with S-O-R theory. On the other hand, introducing uncertainty as a moderating variable to extend S-O-R theory is supported by both previous theory and practice, and there is a practical need to conduct a more in-depth exploration of the underlying mechanism of consumers’ willingness to pay a premium.

### 2.1. Food safety, utilitarian attitudes, and hedonistic attitudes

Attitude is the likelihood that an individual will respond positively or negatively to behavior (Ajzen, 2011), and consumers’ attitudes are considered to be a key element in effectively predicting their behavioral intentions toward organic food (Sadiq et al., 2021). Drawing on Lee and Yun (2015), this paper classifies attitudes into two dimensions based on the function of use and purchase motivation: utilitarian attitudes and hedonistic attitudes. Utilitarian attitudes relate to the functional value of organic food, reflecting the consumer assessment of the benefits when purchasing organic food (Ditlevsen et al., 2019), while hedonistic attitudes relate to emotional satisfaction or sensory experience, reflecting the perceived value derived from the multisensory and emotional aspects of purchasing organic food, such as the taste, freshness, or pleasure derived from doing something good for one’s health and the environment (Wang et al., 2019).

Expectancy-value-attitude theory suggests that an individual’s attitude toward an object is determined by the subjective probability that the object has a particular attribute (Ajzen and Fishbein, 2000). Specifically, an individual will form an overall attitude toward an object by assessing the attributes associated with it and the extent to which its needs are met. Organic food is a credence product that is closely related to consumers’ health and safety and for which product information is difficult to obtain, and there is a certain degree of premium (Yu et al., 2022), so consumers tend to rely on credence attributes for psychological judgment (Janssen and Hamm, 2012; Massey et al., 2018). In organic food consumption, consumers usually pay attention to the food safety of organic food (Vapa-Tankosić et al., 2020; Joya et al., 2021). They believe that organic food is produced without pesticides, chemical fertilizers, artificial additives, and other harmful substances and is safer than ordinary food (Tan et al., 2021; Lang and Rodrigues, 2022), which makes them have a positive attitude toward organic food (Çabuk et al., 2014; Pham et al., 2019). Consumers make positive psychological judgments about food safety that meet their own needs. Such judgments are mainly motivated by the functional and emotional affirmation of organic food, which facilitates the formation of utilitarian attitudes and hedonistic attitudes among consumers. Therefore, the following hypotheses were proposed:

*H1a*: Food safety has a positive effect on utilitarian attitudes.

*H1b*: Food safety has a positive effect on hedonistic attitudes.

### 2.2. Eco-friendliness, utilitarian attitudes, and hedonistic attitudes

Eco-friendliness means that organic food follows the principles of sustainable development in the production process, using specific techniques to avoid genetic engineering and not causing harm to the environment and animals (Honkanen et al., 2006). Information about the sustainability benefits and environmental value of eco-friendliness can influence consumer attitudes toward organic food (Puteri et al., 2022). Consumers will psychologically discern whether the eco-friendliness of organic food meets their needs and further consider whether the pro-environmental characteristics of organic food enhances their personal emotional experience (Gkargkavouzi et al., 2019, Canio et al., 2021). The production process of organic food is concerned with animal welfare and production equity, which promotes ecological balance and makes consumers perceive that buying organic food not only satisfies their own needs but also benefits the ecological environment, which leads to more positive utilitarian attitudes and hedonistic attitudes. Therefore, the following hypotheses were proposed:

*H2a*: Eco-friendliness has a positive effect on utilitarian attitudes.

*H2b*: Eco-friendliness has a positive effect on hedonistic attitudes.

### 2.3. Utilitarian attitudes, hedonistic attitudes, and willingness to pay a premium

The WTPP is the willingness of consumers to pay a higher price for organic food compared to regular food (Konuk, 2018). Consumers’ positive attitudes toward organic food can lead to a greater WTPP (Nassivera et al., 2017). Consumers with a high WTPP are not paying a premium for the organic food itself but for the health and environmental benefits they feel when consuming organic food (Bryła, 2016; Molinillo et al., 2020). This perceived stimulus induces changes in consumers’ emotions and attitudes, which, in turn, affects their WTPP.

According to the theory of planned behavior, attitude is an important antecedent variable of willingness (Yazar and Burucuoğlu, 2019). Attitude reflects not only the individual’s emotional evaluation of a specific thing but also the individual’s behavioral intention. The more positive the individual’s attitude toward a particular thing, the more positive the individual’s intention to develop that behavior (Clark et al., 2019; Salvatore et al., 2021). A positive attitude is an important prerequisite for organic food consumption (Boobalan et al., 2021; Santos et al., 2021). Compared to consumers with a negative attitude, those with a positive utilitarian attitudes and hedonistic attitudes toward organic food believe that organic consumption is beneficial to health and the environment, and this belief not only results in psychological satisfaction but also stimulates their WTPP. Therefore, the following hypotheses were proposed:

*H3a*: Consumers’ utilitarian attitudes have a positive effect on their willingness to pay a premium.

*H3b*: Consumers’ hedonistic attitudes have a positive effect on their willingness to pay a premium.

### 2.4. Mediating role of utilitarian attitudes and hedonistic attitudes

S-O-R theory holds that an individual’s internal psychological state plays a mediating role between external stimuli and behavioral responses; that is, external characteristics stimulate consumers’ behavioral responses by influencing their mental states (Su and Swanson, 2017). Consumers also go through a series of psychological reactions before forming a WTPP for organic food. Specifically, consumers will carefully evaluate the credence attributes of organic food to satisfy their utilitarian and hedonistic goals when purchasing organic food (Sultan et al., 2021). With the external stimulus of the credence attributes of organic food, consumers will develop different attitudes (Brach et al., 2018). On the one hand, the functional value that consumers perceive from the credence attributes of organic food contributes to the development of utilitarian attitudes (Fuljahn and Moosmayer, 2011). On the other hand, consumers may develop hedonistic attitudes because they feel good about doing something that is good for their health and the environment (Maehle et al., 2015). In this case, credence attributes provide consumers a perceived stimulus that influences their attitudes toward organic food and further affects their WTPP. Therefore, the following hypotheses were proposed:

*H4a*: Utilitarian attitudes play a mediating role in the effect of credence attributes and willingness to pay a premium.

*H4b*: Hedonistic attitudes play a mediating role in the effect of credence attributes and willingness to pay a premium.

### 2.5. Moderating role of uncertainty

Uncertainty can be described as a state of incomplete information (Vieira, 2008). When consumers lack reliable information resources and professional knowledge to distinguish organic food, they will doubt the true properties of organic food and generate uncertainty (Yenipazarli, 2015). The current state of information credibility and standards of the organic food market is in a state of confusion, with a variety of certification systems and labels making it difficult for consumers to identify organic food. These potential information asymmetries and ambiguities can increase consumers’ uncertainty (Nuttavuthisit and Thøgersen, 2017) and prevent them from accurately assessing the functional and emotional value of organic food. In this case, uncertainty will reduce consumers’ WTPP even if they have a positive attitude toward organic food (Thøgersen, 2016). Conversely, if the information about the production and control processes of organic food is sufficient, credible, and easily accessible, consumers’ uncertainty about organic food will be reduced, which in turn will lead to a higher WTPP. Therefore, the following hypotheses were proposed:

*H5a*: Uncertainty plays a negative moderating role in the relationship between utilitarian attitudes and willingness to pay a premium.

*H5b*: Uncertainty plays a negative moderating role in the relationship between hedonistic attitudes and willingness to pay a premium.

**Figure 1:**
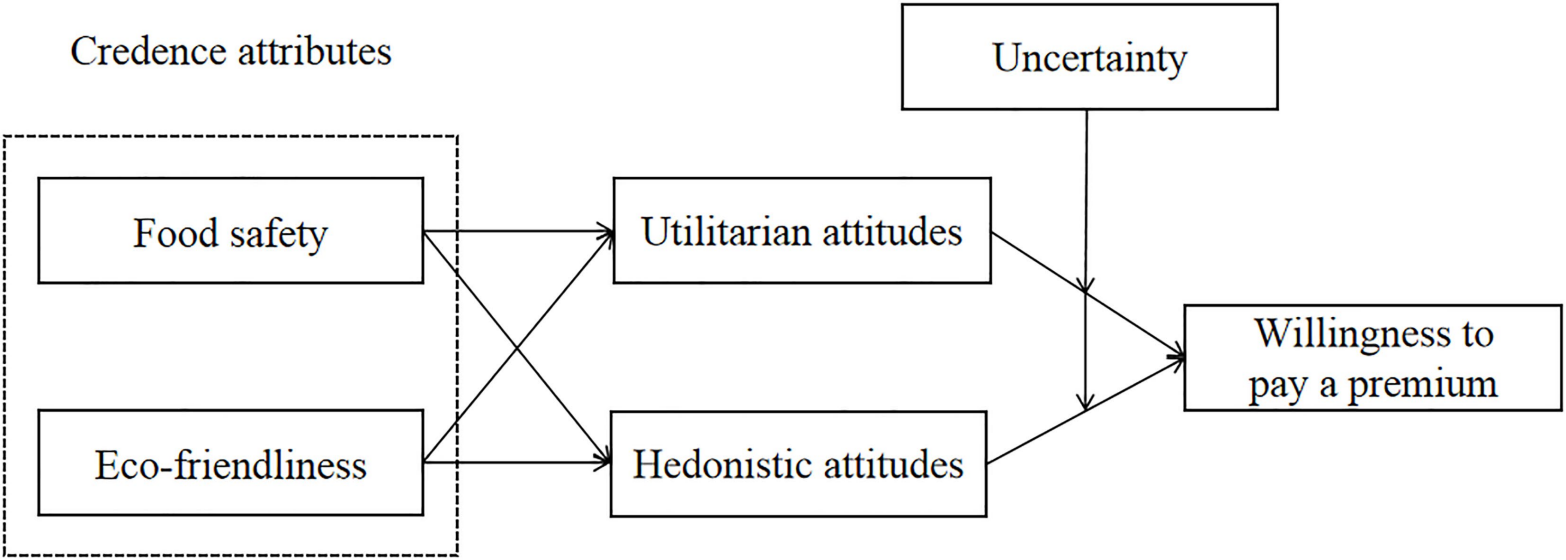
Theoretical model.

Based on the above hypotheses, the theoretical model of this paper is shown in Figure 1.

## 3. Methodology

In methodology, we used descriptive statistics to analyze the profile of the sample. Then we conducted reliability and validity analyses to check the appropriateness of the constructs. Finally, we used structural equation modeling (SEM) to conduct main effects analysis and Process macro in SPSS for mediating and moderating effects analysis.

### 3.1. Sample and procedures

We used the Questionnaire Star platform to design questionnaires. The first part of the questionnaire set up an introductory paragraph introducing organic food and the organic certification label so that participants could fully understand organic food. Two screening questions were also included in the design of the first section. The first was, “Would you like to take part in this survey?” The second was, “Have you ever purchased organic food?” This was done to verify that the participants agreed to participate in the survey and had purchased organic food in order to improve the accuracy of the questionnaire. The second part required participants to answer questions on a 5-point Likert scale regarding credence attributes, attitudes, uncertainty, and willingness to pay a premium, and each page was set to be nonreturnable to prevent participants from repeatedly checking or changing their answers which would invalidate the results of the study. In the third section, participants were asked to fill in some personal information.

Prior to the formal survey, an initial online questionnaire was sent to 10 PhDs in the field of marketing at a large university in Northeast China to ensure that the questions were designed to be easy to understand. Based on their suggestions, unclearly phrased questions were revised and repetitive questions were removed to create the final questionnaire. The formal survey was launched in mid-April. We joined several supermarket discount chat groups and community buying chat groups on the WeChat platform and asked participants to share the online survey among their network of acquaintances using a “snowball” approach to spread the questionnaire online. Given the diversity of organic food buyers, there was no geographical restriction on the distribution of the questionnaires, and a total of 1,000 questionnaires were collected. According to Huang et al. (2015), the presence of insufficient effort responding (IER) in survey data should be assessed prior to hypothesis testing. Therefore, we used response times (Huang et al., 2015; Cheung et al., 2017) to detect and screen out inattentive/careless responding to ensure the reliability of the questionnaire. We tested response times and derived a standard time based on the number of questions, and then excluded questionnaires that took less than the standard time to complete, as well as those with missing data and contradictory questions. A total of 647 valid questionnaires were finally collected.

### 3.2. Measures

The constructs in this study were measured by existing scales adopted from previous studies. Food safety was measured mainly from Steptoe et al. (1995), which contains three measurement items; Eco-friendliness was measured from the Lindeman and Väänänen (2000) four-item scale; Willingness to pay a premium was measured by three question items from Zhang et al. (2020), and the measurement of uncertainty was borrowed from Shiu et al. (2011). These four constructs were measured using a 5-point Likert scale, which classified subjects’ agreement with the question items on seven levels from 1 to 5 (strongly disagree to strongly agree). The utilitarian attitudes and hedonistic attitudes were measured using a scale developed by Voss et al. (2003), each containing four measurement items. The 5-point semantic differential scale was used to classify the subjects’ understanding of the question items on a 5-point scale from 1 to 5. To improve the validity of the questionnaire, opinions were sought from experts in the food and marketing fields, and appropriate modifications were made in the context of the study to ensure that subjects could better understand each question item. The specific question items are shown in Table 1.

**Table 1:** Constructs and items.

Construct	Item	
Food safety	Q1	Organic food contains no additives
	Q2	Organic food contains no artificial ingredients
	Q3	Organic food contains natural ingredients
Eco-friendliness	Q1	The production process of organic food is not harmful to the environment
	Q2	The production process of organic food does not disturb the ecological balance
	Q3	Organic food is packaged in an eco-friendly way
	Q4	The production of organic food does not harm the survival of animals
Utilitarian attitudes	Q1	Non-beneficial - beneficial
	Q2	Unhelpful - helpful
	Q3	Non-functional - functional
	Q4	Unnecessary - necessary
Hedonistic attitudes	Q1	Unpleasant - pleasant
	Q2	Unexciting - exciting
	Q3	Not fun - fun
	Q4	Not thrilling - thrilling
Uncertainty	Q1	I’m not sure that my knowledge of organic food is accurate
	Q2	I’m not sure I’m justified in my view of organic food
	Q3	The organic food label does not allow me to determine if my choice is the best
Willingness to pay a premium	Q1	I am willing to spend more money on organic food than regular food
	Q2	For me, it’s worth buying organic food despite the high price
	Q3	I am willing to pay a premium price for organic food

## 4. Results

### 4.1. Participant demographics

The descriptive statistical characteristics of the sample are specifically shown in Table 2. The results show that there are more women than men among the respondents, accounting for 62.3%, which reflects that the purchasers of organic food in households are mostly women. The age of the respondents is mainly distributed between 20 and 45 years old, and the consumers who buy organic food are mainly young and middle aged. An education level of college, bachelor’s degree, and above accounted for 80.4%, and the monthly income was mainly in the 3,000–6,000 RMB range, accounting for 39.6%, indicating that the average education level and income level of the respondents are relatively high. Overall, the sample has a good representation.

**Table 2:** Demographics of participant (*N* = 647).

Feature	Personal characteristics	Relative frequency	Feature	Personal characteristics	Relative frequency
Gender	Male	37.7%	Education	Senior high school and below	19.6%
	Female	62.3%		Junior college or bachelor degree	58.7%
Age	18–25 years old (including 25 years old)	32.5%		Master degree and above	21.7%
	26–35 years old (including 35 years old)	22.7%	Monthly Income	CNY 3000 and below	39.3%
	36–45 years old (including 45 years old)	23.6%		CNY 3001–6,000 (including CNY 6000)	39.6%
	46–60 years old (including 60 years old)	19.8%		CNY 6001–9,000 (including CNY 9000)	13.4%
	Over 60 years old	1.4%		Over CNY 9000	7.7%

### 4.2. Reliability and validity analysis

Cronbach’s α value was used to test the reliability. As seen in Table 3, the Cronbach’s α values of each construct were above 0.7 (Fornell and Larcker, 1981), and the reliability of the scale was good. Confirmatory factor analysis (CFA) was used to evaluate the validity of the scale, and the results showed that *X*^2^/df = 3.309, less than 5; RMSEA = 0.056, less than 0.08; GFI = 0.928, AGFI = 0.90, CFI = 0.964, NFI = 0.948, IFI = 0.964, all above the benchmark of 0.9, which indicated a good fit of the model and the data (Tabachnick et al., 2007). The standardized factor loadings (estimate) for each item were above the critical value of 0.5. The average variance extracted (AVE) for each construct was above the recommended value of 0.5, and the combined reliability (CR) was above the benchmark of 0.7, implying relatively good convergent validity (Schwab, 2006). Discriminant validity was tested by comparing the AVE with the correlation coefficients of the variables. The square roots of the AVEs on the diagonal in Table 4 were all greater than the correlation coefficients between them and the other constructs, indicating good discriminant validity between the variables (Schwab, 2006).

**Table 3:** Confirmatory factor analysis results.

Construct	Item	Estimate	AVE	CR	Cronbach’s α
Food safety	Q1	0.923***	0.646	0.841	0.823
	Q2	0.866***			
	Q3	0.580***			
Eco-friendliness	Q1	0.858***	0.716	0.910	0.907
	Q2	0.890***			
	Q3	0.765***			
	Q4	0.866***			
Utilitarian attitudes	Q1	0.882***	0.617	0.861	0.838
	Q2	0.930***			
	Q3	0.545***			
	Q4	0.726***			
Hedonistic attitudes	Q1	0.855***	0.730	0.915	0.913
	Q2	0.884***			
	Q3	0.894***			
	Q4	0.780***			
Uncertainty	Q1	0.842***	0.658	0.850	0.838
	Q2	0.912***			
	Q3	0.657***			
Willingness to pay a premium	Q1	0.851***	0.734	0.892	0.894
	Q2	0.887***			
	Q3	0.843***			

***p < 0.001.

**Table 4:** Pearson’s correlations.

	FS	EF	UA	HA	UC	WTPP
FS	0.804					
EF	0.273***	0.846				
UA	0.107***	0.187***	0.785			
HA	0.100***	0.191***	0.400***	0.854		
UC	0.051***	0.120***	0.026**	0.038**	0.811	
WTPP	0.207***	0.377***	0.285***	0.329***	0.076***	0.857

Diagonal values are square roots of average variance extracted (AVE). FS = Food safety; EF = Eco-friendliness; UA = Utilitarian attitudes; HA = Hedonistic attitudes; UC = Uncertainty; WTPP = Willingness to pay a premium. *p < 0.05, ** *p* < 0.01, *** *p* < 0.001.

### 4.3. Hypothesis testing

The hypothesis is tested by the SEM of maximum likelihood estimation. The model fit was *X*^2^ = 418.478, df = 120, *p* < 0.01, *X*^2^/df = 3.487, GFI = 0.934, AGFI = 0.906, CFI = 0.966, NFI = 0.953, IFI = 0.966, RMSEA = 0.062. All fit indicators meet the requirements, indicating a good fit of the model (Browne and Cudeck, 1992). The results based on the structural equation model are shown in Table 5. Food safety had a significant positive influence on both utilitarian attitudes UA (*β* = 0.220, *t* = 2.528, *p* = 0.011) and hedonistic attitudes (*β* = 0.200, *t* = 2.674, *p* = 0.007). Thus, hypotheses H1a and H1b were verified. Eco-friendliness had a significant positive influence on both utilitarian attitudes (*β* = 0.170, *t* = 2.025, *p* = 0.043) and hedonistic attitudes (*β* = 0.220, *t* = 2.603, *p* = 0.009). Thus, hypotheses H2a and H2b were verified. Utilitarian attitudes had a significant positive influence on WTPP (*β* = 0.130, *t* = 3.471, *p* = 0.000). Hedonistic attitudes had a significant positive influence on WTPP (*β* = 0.490, *t* = 11.442, *p* = 0.000). Thus, hypotheses H3a and H3b were verified.

**Table 5:** Results of structural model analysis.

Hypothesized path	*β*	C.R.	*P*
H1a: Food safety → utilitarian attitudes	0.22	2.528	0.011
H1b: Food safety → hedonistic attitudes	0.20	2.674	0.007
H2a: Eco-friendliness → utilitarian attitudes	0.17	2.025	0.043
H2b: Eco-friendliness → hedonistic attitudes	0.22	2.603	0.009
H3a: Utilitarian attitudes → willingness to pay a premium	0.13	3.471	0.000
H3b: Hedonistic attitudes → willingness to pay a premium	0.49	11.442	0.000

### 4.4. Mediation effect test

The mediating effects of utilitarian and hedonistic attitudes were tested using Model 4 (Model 4 is a simple mediation model) in the Process macro proposed by Hayes and Scharkow (2013). The Bootstrap test (a statistical method of multiple repeated sampling) was chosen and set with a number of repetitions of 5,000 and a 95% confidence interval. As shown in Table 6, the bootstrap 95% confidence intervals for both the indirect [0.071, 0.180] and direct [0.386, 0.548] effects of utilitarian attitudes in path 1 did not contain 0, which indicated that utilitarian attitudes partially mediate the relationship between food safety and WTPP. The bootstrap 95% confidence intervals for both the indirect [0.068, 0.169] and direct [0.405, 0.562] effects of utilitarian attitudes in path 3 did not contain 0, which indicated that utilitarian attitudes play a partially mediating role in the relationship between eco-friendliness and WTPP. Hypothesis H4a was verified. The bootstrap 95% confidence intervals for both the indirect [0.103, 0.218] and direct [0.353, 0.511] effects of hedonistic attitudes in path 2 did not contain 0, which indicated that hedonistic attitudes play a partially mediating role in the relationship between food safety and WTPP. In path 4, the bootstrap 95% confidence intervals for both the indirect [0.010, 0.207] and direct [0.372, 0.525] effects of hedonistic attitudes did not contain 0, which indicated that hedonistic attitudes play a partially mediating role in the relationship between eco-friendliness and WTPP. Thus, hypothesis H4b was verified.

**Table 6:** Results of mediation analysis.

Path	Direct effect		Indirect effect	
	*β*	Bias-corrected 95% CI	*β*	Bias-corrected 95% CI
FS → UA → WTPP	0.432	[0.386, 0.548]	0.156	[0.071, 0.180]
FS → HA → WTPP	0.467	[0.353, 0.511]	0.121	[0.103, 0.218]
EF → UA → WTPP	0.484	[0.405, 0.562]	0.114	[0.068, 0.169]
EF → HA → WTPP	0.449	[0.372, 0.525]	0.149	[0.010, 0.207]

FS = Food safety; EF = Eco-friendliness; UA = Utilitarian attitudes; HA = Hedonistic attitudes; WTPP = Willingness to pay a premium.

By comparing the relative magnitude of the mediating effect, in the indirect relationship between food safety and WTPP, the proportion of the mediating effect of utilitarian attitudes is 0.156/0.588 = 26.53% and the proportion of the mediating effect of hedonistic attitudes is 0.121/0.588 = 20.58%, indicating that the mediating effect of utilitarian attitudes is greater than that of hedonistic attitudes. However, in the indirect relationship between eco-friendliness and WTPP, the proportion of the mediating effect of utilitarian attitudes is 0.114/0.598 = 19.06%, and the proportion of the mediating effect of hedonistic attitudes is 0.149/0.598 = 24.91%, indicating that the mediating effect of hedonistic attitudes is greater than that of utilitarian attitudes.

### 4.5. Moderating effect test

The moderated mediation analysis model (Process Model 14) proposed by Hayes (2018) was used to test for moderating effects and mediating effects with moderation. Food safety and eco-friendliness were placed into the model as independent variables, utilitarian and hedonistic attitudes as mediating variables, uncertainty as a moderating variable, and WTPP as a dependent variable. The results of the test for the moderating effect of uncertainty (Table 7) show that uncertainty plays a significant negative interaction effect on the relationship between utilitarian attitudes and WTPP (*β* = −0.185, *p* = 0.012; *β* = −0.186, *p* = 0.011). Thus, hypothesis H5a was verified. Uncertainty plays a significant positive interaction effect on the relationship between hedonistic attitudes and WTPP (*β* = 0.132, *p* = 0.040; *β* = 0.138, *p* = 0.030). Thus, hypothesis H5b was not verified.

**Table 7:** Results of the moderating effect test.

Independent variable	Interaction items	*β*	Boot SE	*t*	*p*
Food safety	Utilitarian attitudes * Uncertainty	−0.185	0.074	−2.518	0.012
	Hedonistic attitudes * Uncertainty	0.132	0.064	2.056	0.040
Eco-friendliness	Utilitarian attitudes * Uncertainty	−0.186	0.073	−2.548	0.011
	Hedonistic attitudes * Uncertainty	0.138	0.063	2.180	0.030

The test for mediating effects with moderation is shown in Table 8. When the independent variable was food safety, the moderating effect of low-uncertainty consumers on the mediating effect of utilitarian attitudes was significant (indirect effect = 0.007, boot CI = [0.029, 0.140]); the moderating effect of high-uncertainty consumers on the mediating effect of utilitarian attitudes was not significant (indirect effect = −0.015, boot CI = [−0.080, 0.067]). The results indicate that there is a significant difference between the low-uncertainty and high-uncertainty groups in terms of whether they influence the WTPP through utilitarian attitudes. The mediating effect of utilitarian attitudes is moderated by uncertainty. In addition, according to the determination index, it can be further shown that uncertainty strengthens the mediating role of utilitarian attitudes in food safety and WTPP (index = −0.069, boot CI = [−0.140, −0.003]).

**Table 8:** Mediating effects with moderation.

Independent variable	Intermediate variables	Regulating variables	Effect	Boot CI	Index	Boot CI
Food safety	Utilitarian attitudes	Low uncertainty	0.077	[0.029, 0.140]	−0.069	[−0.140, −0.003]
		High uncertainty	−0.015	[−0.080, 0.067]		
	Hedonistic attitudes	Low uncertainty	0.100	[0.042, 0.160]	0.056	[0.001, 0.121]
		High uncertainty	0.174	[0.106, 0.251]		
Eco-friendliness	Utilitarian attitudes	Low uncertainty	0.074	[0.029, 0.138]	−0.065	[−0.138, −0.001]
		High uncertainty	−0.013	[−0.075, 0.067]		
	Hedonistic attitudes	Low uncertainty	0.092	[0.035, 0.152]	0.057	[0.001, 0.121]
		High uncertainty	0.168	[0.104, 0.243]		

The high-uncertainty group (indirect effect = 0.174, boot CI = [0.106, 0.251]) moderated the mediating effect of hedonistic attitudes more than the low-uncertainty group (indirect effect = 0.100, boot CI = [0.042, 0.160]), and there was a significant difference in the indirect effect between the two groups, indicating that the mediating effect of hedonistic attitudes was moderated by uncertainty. In addition, uncertainty strengthened the mediating role of hedonistic attitudes between food safety and WTPP (index = 0.056, boot CI = [0.001, 0.121]). Similarly, when the independent variable is eco-friendliness, uncertainty strengthens the mediating role of utilitarian attitudes and hedonistic attitudes between eco-friendliness and WTPP.

## 5. Discussion and conclusion

We found that the credence attributes of organic food, as a psychological evaluation of consumers after receiving information about organic food, significantly and positively influence consumers’ utilitarian and hedonistic attitudes. This result is consistent with previous studies that found that health, safety, and sustainability motivations (environmental factors) positively influence consumer attitudes toward organic food (Pham et al., 2019; Rana and Paul, 2020). Based on these studies, our research focused on two attributes—food safety and eco-friendliness—and further found that consumers’ perceived functional value comes mainly from the food safety of organic food, which is more likely to stimulate consumers’ utilitarian attitudes and influence their WTPP compared to eco-friendliness. Eco-friendliness has pro-environmental characteristics and can enhance consumers’ emotional experience compared to food safety, so eco-friendliness is more likely to stimulate consumers’ hedonistic attitudes and thus promote their WTPP.

Lee and Yun (2015) showed that utilitarian and hedonistic attitudes have a significant positive effect on behavioral intention to purchase organic food and that utilitarian attitudes have a greater impact on consumers’ purchase intention than hedonistic attitudes, with consumers tending to approach organic purchase intention with utilitarian attitudes. Our research also found that utilitarian and hedonistic attitudes positively influence consumers’ WTPP, meaning that consumers are looking not only for superior functional value when buying organic food but also for an emotional experience of consumption. However, we found that hedonistic attitudes had a greater impact on consumers’ WTPP than utilitarian attitudes, which may be due to the fact that consumers react differently to the willingness to pay a premium price (WTPP) versus the willingness to buy (WTB). When the consumer has decided to purchase organic food and is then confronted with a high price, hedonistic attitudes become more prominent in the decision-making process.

In this study, we found that utilitarian and hedonistic attitudes partially mediate the effect of credence attributes on consumers’ WTPP. In other words, food safety and eco-friendly attributes have a significant direct effect on WTPP, while they are also mediated indirectly by utilitarian and hedonistic attitudes. Lee et al. (2019) found that utilitarian and hedonistic attitudes mediated the relationship between factors related to credence attributes such as healthy content, local production, and organic food labels on purchase behavior. In fact, consumers make positive cognitive and emotional judgments about the credence attributes associated with organic food, such as associating organic food with safety and health and ecological conservation (Knaggs et al., 2022), which makes them aware of the functional and emotional benefits and value of organic food, thus increasing their WTPP.

Both Teng and Lu (2016) and Nuttavuthisit and Thøgersen (2017) showed in their studies that high levels of uncertainty reduce consumers’ willingness to purchase organic food. This differs somewhat from our study, where we argue that the effect of uncertainty on consumers’ behavioral intentions depends on consumers’ attitudes. We found that uncertainty negatively moderates the effect of utilitarian attitudes on the WTPP and positively moderates the effect of hedonistic attitudes on the WTPP. This result suggests that perceived risk due to uncertainty negatively affects utilitarian consumers’ WTPP, meaning that utilitarian consumers are less likely to make the decision to pay a premium price when they feel uncertain about information related to organic food, as they do not have the relevant knowledge or information to accurately predict the outcome of the transaction (Hassan et al., 2013). For hedonistic consumers, uncertainty does not reduce their WTPP, probably because most consumers with hedonistic attitudes are less price sensitive and more willing to take risks when making hedonistic purchases (Sadiq et al., 2021), so they may also be willing to pay a premium for organic food in the face of uncertainty because of the emotional satisfaction that buying organic food can bring them. In addition, we further found that uncertainty moderates the mediating role of utilitarian and hedonistic attitudes between credence attributes and WTPP, suggesting that the difference in indirect effects is significant at high and low levels of uncertainty.

### 5.1. Theoretical implications

The foremost theoretical contribution of this research is the extension of the S-O-R model in the context of organic food consumption. Although S-O-R models have been used for predicting purchase intentions of organic food (Kim et al., 2021; Sultan et al., 2021), the S-O-R framework in the organic food context is currently still underrepresented in the literature, especially in the Chinese context. Moreover, there is still a lack of studies to measure other causal and moderating factors in the framework (Lee and Yun, 2015), so a more robust exploration of the framework is necessary. This study is among the first to develop a model that explains how consumers’ willingness to pay a premium for organic food is driven and constrained by integrating food safety, eco-friendliness, utilitarian attitudes, hedonistic attitudes, and uncertainty. It extends the S-O-R theory and its application context. At the same time, the results of the study provide a new theoretical basis for companies to develop organic food marketing strategies.

Secondly, this study reveals the internal psychological mechanisms involved in the decision-making process of consumers in purchasing organic food. While a large number of studies have highlighted the role of attitudes in intention to purchase organic food and actual purchase behavior (Boobalan et al., 2021; Santos et al., 2021), they have mainly focused on unidimensional attitudes (Lee and Goudeau, 2014). This research investigated utilitarian and hedonic dimensions of attitudes toward purchasing organic foods. This conceptualization is a more integrative approach than the previous studies in which one-dimensional concept of attitudes is considered. Further, the findings suggest that hedonistic attitudes have a greater impact on consumers’ WTPP, implying that consumers may be more inclined to seek a superior emotional experience when purchasing organic food, which will provide new dimensions of exploration for future research in the area of consumer attitudes and behavior.

Thirdly, this study strengthens the explanatory power of the S-O-R model by adding uncertainty as a moderating variable to the model. Our study identifies the moderating role of uncertainty in the relationship between different dimensions of attitudes and WTPP, contributing to the explanation of the attitude-behavior gap in organic food research. Furthermore, our study found that uncertainty strengthens the mediating role of utilitarian and hedonistic attitudes between credence attributes and WTPP. This moderated mediating role could provide new and substantial insights into marketing theory and future research directions and could expand the boundaries of existing organic food research. Finally, the study spotlights the behavior of Chinese consumers in regard to organic food. Findings bridge the gap of deficient theoretical knowledge on the unique characteristics of Chinese consumers and their responses to organically grown food items.

### 5.2. Managerial implications

Firstly, we suggest that manufacturers could consider carefully designing safety and environmental messages to showcase how organic food contributes to the health and well-being of consumers. Specifically, in terms of eco-friendliness, manufacturers may consider reporting the carbon footprint of their products and creating a consumer sustainability index (Nikolaou and Kazantzidis, 2016) to improve the perceived environmental value of a product among consumers. In terms of food safety, retailers could cite the latest and most authoritative scientific findings or endorsements by national agencies to support their claims on the safety of organic food, such as less additives and pesticide residues, more beneficial nutrients, etc. In addition, retailers could communicate the safety and environmental benefits of organic foods through social networks, mass media, and interactive screens in-store, and include integrated promotional mix elements in their communication campaign (Chekima et al., 2019).

Secondly, as established consumer attitudes are difficult to change (Aaker, 1996), we suggest that marketers could stimulate positive attitudes toward organic food by providing consumers with cues that organic food contains hedonic and utilitarian attributes that will satisfy their needs. Specifically, for utilitarian consumers, marketers may participate or sponsor research related to the organic food safety testing and reflect the findings in advertising, packaging, or leaflets, which will help stimulate utilitarian attitudes among consumers. For hedonistic consumers, marketers could focus on hedonic gratification derived from purchasing organic foods. For example, by emphasizing that an organic food purchase is a way to ultimately save animals and the environment, or by using experiential marketing methods, organizing food tastings, food festival competitions, etc. to stimulate hedonic feelings such as satisfaction. According to our findings, hedonistic attitudes have a stronger impact on consumers’ WTPP compared to utilitarian attitudes. Therefore, marketers could leverage the hedonic benefits of the consumption of organic food by engaging in emotional appeal advertising and highlight the hedonic attributes of their products through a multi-sensory marketing approach (attractive packaging and good taste, etc.), thus enhancing consumer pleasure and enjoyment.

Finally, we recommend that manufacturers may consider introducing the latest blockchain technology. Blockchain food traceability system has the characteristics of traceability and tamper-proof, which can ensure the authenticity of information (Wei et al., 2022), thus reducing the perceived information asymmetry between consumers and organic food suppliers. Manufacturers could also introduce QR codes with traceability information on product packaging. By scanning the QR code, consumers could have information on the entire process of organic products from production to distribution, which will help increase transparency and address consumers’ uncertainty. In addition, organic producers could show more production details to consumers through media and social platforms to reduce consumers’ uncertainty and increase their organic purchase intentions.

## 6. Limitations and future directions

One limitation of this study is that participants were recruited from supermarket discount chat groups and community buying chat groups on WeChat. There were more participants who were female and aged 20–45 years old, which may not be generalizable. Future studies could expand the sample set in terms of gender and age so that the findings may be more valid. Another limitation is that the sample in this study was not stratified by place of purchase. Future studies could divide the sample set into different segments such as organic food supermarkets, specialty shops, and bazaars. In addition, this paper is an exploration of the willingness to pay a premium for organic food, and future research could further explore consumers’ willingness to repurchase organic food.

## Data availability statement

The raw data supporting the conclusions of this article will be made available by the authors, without undue reservation.

## Ethics statement

Ethical review and approval were not required for the study on human participants in accordance with the local legislation and institutional requirements. Written informed consent for participation was not required for this study in accordance with the national legislation and institutional requirements.

## Author contributions

HH: conceptualization. XJ and CH: data curation. XJ: formal analysis. HH and SW: funding acquisition. DY and YT: investigation. CH: methodology. HH and CH: resources. XJ and YT: writing—original draft. SW and DY: writing—review and editing. All authors contributed to the article and approved the submitted version.

## Funding

This study was funded by the reform and develop high-level talent projects in local universities supported by the central government (2020GSP13); National Social Science Fund of China (22BJY157); Natural Science Foundation of Heilongjiang Province (LH2022G014); Social Science Foundation Project of Heilongjiang Province (20GLB114); and Postdoctoral research start-up fund project of Heilongjiang Province (LBH-Q21103).

## Conflict of interest

The authors declare that the research was conducted in the absence of any commercial or financial relationships that could be construed as a potential conflict of interest.

## Publisher’s note

All claims expressed in this article are solely those of the authors and do not necessarily represent those of their affiliated organizations, or those of the publisher, the editors and the reviewers. Any product that may be evaluated in this article, or claim that may be made by its manufacturer, is not guaranteed or endorsed by the publisher.
